# Supplementary material for: Minor Glomerular Abnormalities are Associated with Deterioration of Long-Term Kidney Function and Mitochondrial Injury
Source: J Clin Med. 2019 Dec 22;9(1):33. doi: 10.3390/jcm9010033 (PMC7019622; doi:10.3390/jcm9010033)
Supplement: Supplementary file 1 [file jcm-09-00033-s001.pdf]

Journal of
Clinical Medicine

MDPI

Supplementary Materials

Table S1. Changes in the eGFRs of the MGA, MHC, and IgAN groups in the retrospective study.

Variable	MHC Group (*n* = 49)	MGA Group (*n* = 49)	*p* Value[a]	IgAN Group (*n* = 49)	*p* Value[b]
Mean eGFR (mL/min/1.73 m^2)					
Baseline	97.04 ± 17.41	98.13 ± 18.30	0.761	98.48 ± 17.31	0.924
After 1 year	96.57 ± 18.98	87.08 ± 17.58	0.054	90.92 ± 18.79	0.394
After 2 years	94.63 ± 18.28	90.21 ± 19.92	0.419	88.93 ± 19.68	0.790
After 3 years	92.78 ± 17.99	81.54 ± 18.63	0.063	87.77 ± 18.71	0.262
After 4 years	95.36 ± 15.55	77.47 ± 15.80	**0.002**	84.63 ± 17.64	0.159
After 5 years	90.92 ± 16.91	75.73 ± 17.27	**0.005**	81.89 ± 22.56	0.335
After 10 years	89.82 ± 16.75	61.37 ± 15.71	**<0.001**	75.26 ± 17.40	**0.015**

Data are expressed as the mean ± standard deviation. [a]MGA group and MHC group; [b]MGA group and IgAN group. MGA, minor glomerular abnormality; MHC, matched heathy control; IgAN, immunoglobulin A nephropathy; eGFR, estimated glomerular filtration rate.

Table S2. MEST-C score of retrospective and prospective IgAN cohorts.

Variables	Rectrospective IgAN cohort (*n* = 49)	Prospective IgAN cohort (*n* = 15)	*p*-value
Oxford classification			
M score 1	26 (53.1%)	9 (60.0%)	0.637
E score 1	2 (4.1%)	3 (20.0%)	0.079
S score 1	17 (34.7%)	9 (60.0%)	0.081
T score 1	18 (36.7%)	5 (33.3%)	0.810
T score 2	1 (2.0%)	0 (0.0%)	> 0.999
C score 1	4 (8.2%)	3 (20.0%)	0.340
C score 2	0 (0.0%)	0 (0.0%)	-

Data are expressed as *n* (%) for categorical variables. IgAN, immunoglobulin A nephropathy; M, mesangial hypercellularity; E, endocapillary proliferation; S, segmental glomerulosclerosis; T, tubular atrophy/interstital fibrosis; C, cellular/fibrocellular crescent.

Table S3. Comparison of tranditional prognostic markers at presentaion between low and high mtDNA. Groups in patients with minor glomerular abnormalities.

Variables	Low mtDNA group (*n* = 7)	High mtDNA group (*n* = 8)	*p*-value
eGFR (mL/min/1.73 m^2)	100.50 ± 8.08	106.62 ± 15.97	0.397
Proteinuria (mg/24 h)	641.67 ± 1040.87	473.97 ± 342.87	0.397
Mean arterial pressure (mmHg)	93.24 ± 8.30	79.58 ± 8.81	0.009

Data are shown as mean ± standard deviation for continuous variables and were analyzed by Mann-Whitney.

U-tests. mtDNA: mitochondrial DNA, eGFR: estimated glomerular filtration rate.

Table S4. Baseline characteristics of the MGA, MHC, and IgAN groups in both retrospective and prospective studies.

Variable	MHC Group (*n* = 64)	MGA Group (*n* = 64)	*p* Value[a]	IgAN Group (*n* = 64)	*p* Value[b]
Age (years)	31.08 ± 12.88	30.73 ± 14.43	0.887	30.66 ± 11.89	0.973
Sex (male)	43 (67.2%)	43 (67.2%)	>0.999	43 (67.2%)	>0.999
Body mass index (kg/m^2)	22.87 ± 3.68	24.59 ± 4.49	0.157	24.03 ± 4.29	0.474
Diabetes	0 (0.0%)	1 (1.6%)	>0.999	2 (3.1%)	>0.999
Hypertension	0 (0.0%)	7 (10.9%)	**0.013**	12 (18.8%)	0.214
Systolic blood pressure (mmHg)	116.64 ± 10.15	123.84 ± 12.77	**0.001**	126.98 ± 13.26	0.175
Diastolic blood pressure (mmHg)	74.63 ± 7.51	75.88 ± 11.06	0.465	80.58 ± 9.70	**0.012**
Mean arterial pressure (mmHg)	87.21 ± 13.58	91.86 ± 10.58	**0.034**	96.05 ± 9.94	**0.023**
Baseline SCr levels (mg/dL)	0.96 ± 0.18	0.96 ± 0.20	0.864	1.03 ± 0.26	0.088
Baseline eGFR (mL/min/1.73 m^2)	99.63 ± 16.11	99.45 ± 17.25	0.860	93.58 ± 19.70	0.075
Baseline proteinuria (mg/day)	ND	789.19 ± 796.61	ND	1129.8 ± 1145.24	**0.037**
Use of ARB or ACE inhibitor	ND	29 (45.3%)	ND	54 (84.4%)	**<0.001**
Use of immunosuppressant	ND	13 (20.3%)	ND	16 (25.0%)	0.526
Urinary RBC (counts/HPF)		27.60 ± 38.14	ND	22.24 ± 28.76	0.371
Reasons for kidney biopsy					**0.040**
Isolated hematuria		11 (17.2%)		6 (9.4%)	
Isolated proteinuria		21 (32.8%)		12 (18.8%)	
Both hematuria and proteinuria		32 (50.0%)		46 (71.9%)	

Data are expressed as the mean ± standard deviation for continuous variables and *n* (%) for categorical variables. [a]MGA and MHC groups; [b]MGA and IgAN groups. MHC, matched healthy control; MGA, minor glomerular abnormality; IgAN, immunoglobulin A nephropathy; SCr, serum creatinine; eGFR, estimated glomerular filtration rate; ARB, angiotensin II receptor blocker; ACE, angiotensin converting enzyme; ND, not determined; RBC, red blood cells; HPF, high power field.

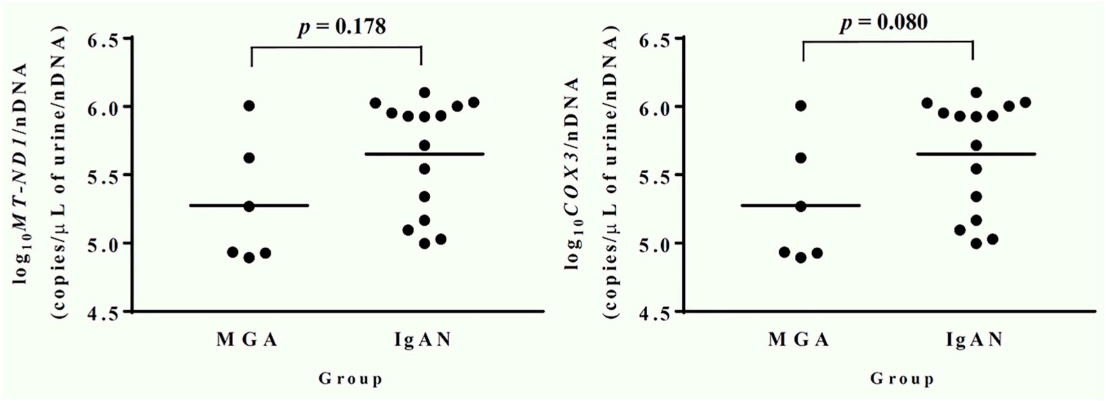

Figure S1. Comparison of urinary mitochondrial DNA copy numbers in the minor glomerular abnormality (MGA) and immunoglobulin A nephropathy (IgAN) groups who were treated with angiotensin II receptor blocker or angiotensin converting enzyme inhibitor. Data were analyzed by the Mann-Whitney test. *MT-ND1*, mitochondrially encoded NADH dehydrogenase 1; *COX3*, cytochrome c oxidase subunit III.

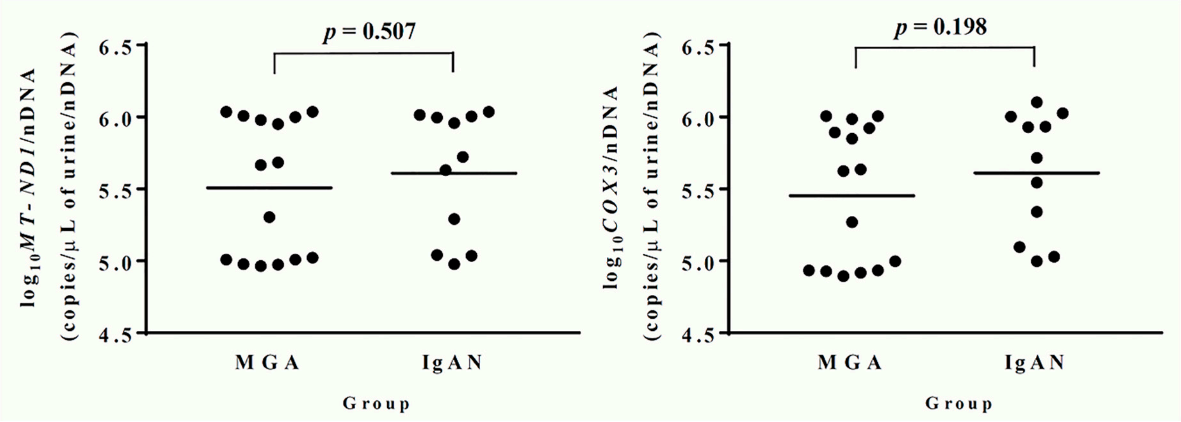

Figure S2. Comparison of urinary mitochondrial DNA copy numbers in the minor glomerular abnormality (MGA) group and patients with immunoglobulin A nephropathy (IgAN) without endocapillary proliferation and crescent formation. Data were analyzed by the Mann-Whitney test. *MT-ND1*, mitochondrially encoded NADH dehydrogenase 1; *COX3*, cytochrome c oxidase subunit III.

© 2019 by the authors. Licensee MDPI, Basel, Switzerland. This article is an open access article distributed under the terms and conditions of the Creative Commons Attribution (CC BY) license (http://creativecommons.org/licenses/by/4.0/).
